# Flattening the quality of life curve? A prospective person-centred study from Norway amid COVID-19

**DOI:** 10.1007/s11136-022-03113-2

**Published:** 2022-03-24

**Authors:** Ragnhild Bang Nes, Baeksan Yu, Thomas Hansen, Øystein Vedaa, Espen Røysamb, Thomas S. Nilsen

**Affiliations:** 1 Department of Mental Health and Suicide, The Norwegian Institute of Public Health, Oslo, Norway; 2 Promenta Research Centre, Department of Psychology, University of Oslo, Oslo, Norway; 3 Department of Child Health and Development, The Norwegian Institute of Public Health, Oslo, Norway; 4 Department of Health Promotion, Norwegian Institute of Public Health, Bergen, Norway; 5 Department of Mental Health, Norwegian University of Science and Technology, Trondheim, Norway; 6 Voss District Psychiatric Hospital, NKS Bjørkeli, Voss, Norway; 7 Department of Research and Development, St. Olavs University Hospital, Trondheim, Norway

**Keywords:** Quality of life, Subjective wellbeing, Depression, Anxiety, COVID-19, Latent transition analysis

## Abstract

**Purpose:**

We examined multidimensional, heterogeneous reactions to the COVID-19 pandemic and associated measures to provide further insights into the developmental processes of risk and adaptation.

**Method:**

We used three-wave questionnaire data from 8156 individuals participating in the Norwegian County Public Health Survey assessed 1–5 months before and three (June 2020) and nine (December 2020) months after the outbreak. Latent profile and latent transition analyses were used to identify latent quality of life (QoL) classes and multiform changes, their probabilities, and predictors.

**Results:**

We identified five distinct QoL classes of varying proportions, namely *Flourishing* (i.e. 24–40%), *Content* (31–46%), *Content-Symptomatic* (8–10%), *Languishing* (14–20%), and *Troubled* (2–5%). Despite higher levels of negative affect and lower levels of life satisfaction and positive emotions, most individuals remained in their pre-pandemic QoL profiles. Yet, changes occurred for a meaningful proportion, with transition to a less favourable class more common than to a favourable. Between time 1 and 3, the flourishing and troubled groups decreased by 40% and 60%, while the content and languishing groups increased by 48% and 43%, respectively. Favourable pre-pandemic relational (marital status, support, interpersonal trust, and belonging), health, and economy-related status predicted significantly lower odds of belonging to the high-risk groups both pre-pandemic and during the pandemic.

**Conclusions:**

Overall, this study shows lower levels of QoL amid the COVID-19 pandemic, but substantial stability in the QoL distribution, and an overall levelling of the QoL distribution. Our findings also underscore the importance of financial, health-related, and social capital to QoL.

**Supplementary Information:**

The online version contains supplementary material available at 10.1007/s11136-022-03113-2.

Major life events, community-wide disasters, and macrolevel changes often compromise mental health and quality of life (QoL), particularly when posing an immediate threat and restricting social, economic, and work-related aspects of life [1, 2]. On March 11, 2020, the World Health Organization (WHO) declared the novel coronavirus disease 2019 (COVID-19) a global pandemic. Since then, the pandemic has posed unprecedented challenges to healthcare systems and affected the global population through the direct/indirect costs of the disease as well as the governmental restrictions imposed to mitigate its spread and impact. The pandemic is increasingly regarded as a mental health crisis of unparalleled scope and scale, with the consequences likely to be profound, wide-reaching, and long-lasting, not least because of a potential fallout of an economic downturn [3–6]. The pandemic is indicated to substantially amplify social inequalities and health inequity, further aggravating the risk for long-term mental health problems [7]. Excess rates of anxiety, depression, loneliness, and stress have been firmly documented, especially in the early and middle stages of the pandemic [8–14], with emerging evidence showing both exacerbation of pre-existing mental health problems [15, 16] and new symptoms in individuals with no previous disorders [17]. The mental health impact appears to disproportionately affect disadvantaged groups, including women, young people, those suffering from chronic/psychiatric illnesses, and those having poor income [18], with inequalities in mental health rising with pandemic severity levels [19].

Against this bleak picture, a number of studies suggest a less alarming situation [20]. For example, data from the Gallup World Poll show significant, yet only small increases in the frequency of negative emotions globally in 2020 and no changes in positive emotions [21]. Several factors may explain this stability, with one important factor being human adaptation. There is substantial stability in both positive and negative emotion partly due to affective equilibrium levels maintained by personality, genetic influences, and enduring environmental circumstances [22–25]. Stressful events often cause fluctuations beyond the habitual emotional range, but over time a return to pre-event levels tends to occur by means of biological (e.g. genetic), cognitive (e.g. relabelling, benefit-finding, and downward adjustment), and behavioural (e.g. coping strategies) mechanisms—albeit with less adaptation to ongoing stressors [26, 27].

Another important factor relates to most challenges and stressors also affording upsides or mixed reactions. For example, crises tend to produce groundswells of prosocial behaviour, leading to positive both individual and collective outcomes (e.g. solidarity, belonging), with “catastrophe-compassion” found to be widespread and consistent [28]. A nationally representative Norwegian QoL survey indicated higher distress levels during the March 2020 lockdown, but also greater social support [29]. In a UK survey in April 2020, 68% reported increased kindness and 40% felt more connected to others in their community, with significantly higher QoL and lower depression reported by these individuals [30]. Likewise, 62% of New Zealanders also surveyed in April 2020 reported pandemic-related ‘silver linings’ including working from home, spending more time with their family, and enjoying a more quiet, less polluted environment [31]. Reduced loneliness [32], increased meaning and optimism [33], lower prevalence of mental disorders, and stable levels of suicidal ideation and suicide deaths [34] have also been documented, at least during the initial stages of the pandemic.

A third factor pertains to methodological limitations that question the validity and utility of many published findings [35]. For example, few studies employ a robust prospective design with baseline assessments prior to the outbreak. Many studies are restricted by non-representative samples or modest sample sizes and most have not included measures of mental strengths. When focussing narrowly on symptoms and disorders, key emotional experiences (e.g. joy and meaning) of significance to thriving and adaptation are easily ignored. Positive and negative indicators seem to lie on partly separate continua [23, 36] and the absence of distress is not sufficient for high QoL [37]. Most studies also use a variable-centred approach, assuming a single, homogeneous population rather than multiple subpopulations (i.e. person-centred), despite the pandemic likely to cause mixed experiences across different groups.

By examining a broader range of both symptoms (e.g. anxiety, worry, and sadness) and assets (e.g. joy, meaning, and satisfaction) over time and investigating different subgroups, we may capture a greater range of heterogeneity [38, 39], provide a more holistic understanding of the QoL impact of the pandemic, and better identify modifiable factors that predict healthy and unhealthy adjustment.

To examine changes over time and address the abovementioned gap pertaining to the heterogeneity and complexity of reactions to COVID-19, we use three-wave data from a large, prospective Norwegian sample assessed before and at three and nine months into the pandemic. We apply latent profile (LPA) and latent transition (LTA) analysis to examine multidimensional and heterogenous reactions. We particularly investigate (i) QoL levels before and during the pandemic, (ii) the number and composition of distinct QoL profiles (i.e. clinically meaningful subgroups), (iii) transition patterns across these profiles during the COVID-19 pandemic, and (iv) social, health-related, and demographic baseline predictors to identify the attributes of vulnerable and stress-resistant individuals/subgroups.

We expect the pandemic to have negatively affected QoL levels. National lockdown and a wide range of non-pharmacological interventions were introduced in Norway on March 12, 2020, many have remained over extended time, and substantially infringed on personal freedom, restricted social life, affected financial security, and limited regular sources of wellbeing. The consequences of the virus have thus not only been physical (e.g. illness, hospitalization, sedentary life style) and financial (e.g. redundancy, financial insecurity) [40], but also socio-emotional (e.g. fear, loneliness, isolation), causing a sharp increase in most putative risk factors for poor QoL. We also expect to identify different QoL profiles, varying not only in their levels but also in their composition. As QoL is substantially influenced by personality and genetic influences [22, 25, 41], we hypothesize a fairly stable QoL distribution. Yet, as both positive and negative indicators of QoL tend to change in response to abrupt events and ongoing stressors [27], we expect some transition towards the lower QoL class and hypothesize that pre-pandemic social integration, good health, and higher socio-economic status relate to better QoL also during the pandemic.

## Method

### Sample

The Norwegian Counties Public Health Survey (NCPHS) is a cross-sectional survey used for monitoring and identification of locally salient health determinants. Invitations to the online NCPHS are distributed by email/SMS. Email addresses and cell phone numbers to eligible participants were retrieved from the national registers of the Norwegian Digital Agency. Baseline data (T1) for the present study are the NCPHS collected in Agder (23 September–18 October 2019, *N* = 28,047, response rate (RR) = 46%) and Nordland (27 January–16 February 2020, *N* = 24,222, RR = 47%) counties. A random sample of 20,196 from these two counties was invited to participate in a longitudinal NCPHS COVID-19 study (T2). These two counties participated in the NCHPS close in time (< 6 months) to the national COVID-19 lockdown (12 March 2020). Data for the second wave were collected 4–18 June 2020 (*N* = 11,953, RR = 59% of those invited) and a third assessment was conducted 19 November–4 December 2020 (T3). Of the initial sample, 30% dropped out from later assessments. Altogether 8,156 individuals participated in all the three assessments and constituted our target sample. Compromised health, lower education, financial difficulties, and younger age significantly predicted drop-out from T1. However, subsequent drop-out status (i.e. using the full T1 sample) explained only 1% of the variance in the outcome variables at T1.

### Measures

The NCPHS includes questions on QoL, health, and community factors. The QoL items constitute a national standard developed to provide local, regional, and national authorities with holistic, comparable steering information to guide policy development and evaluate societal changes [42]. The questions included one item on life satisfaction (LS), one on meaning in life, and six items on the presence of positive emotions (PE: happy, calm, and engaged) and negative emotions (NE: sad/blue, worried, and anxious) the last week. All eight items correspond to monitoring items used/recommended by the Office of National Statistics [43] and the OECD [44], and all are scored on a 0–10 scale (0 = “not at all”, 10 = “very much”) in accordance with OECD guidelines [44]. Generally, single-item LS and meaning measures perform very similar to multiple-item scales [45, 46]. A previous Confirmatory Factor Analysis of the LS item and the five Satisfaction With Life Scale items [47, 48] yielded one factor and the LS item loaded 0.75 on this factor. Cronbach’s Alphas for the three-item NE and PE measures were estimated to 0.84 and 0.71, respectively. Mean values were used, and kurtosis and skewness of the four dependent variables ranged from 2.50 to 4.50 and − 1.11 to 0.71, respectively. Variables were then standardized to zero mean and unit variance and then entered in LPA/LTA.

The baseline predictors included *sex* (male = 1, female = 0), *age* (continuous), *subjective income* (0 = “very difficult” to 5 = “very easy”), *educational level* (“high school”, “2–3 years college”, “4 years college”), *marital status* (“married/cohabitating”, “non-resident partner”, “single”), *employment status* (“employed”, “disabled/unemployed”, “other” [student, military service, retired]), *self-rated health* (0 = “very poor” to 4 = “very good”), *interpersonal trust* (scale 0–10), and *sense of belonging* (scale 0–10). *Social support* was measured with the Oslo Support Scale (OSS-3 [49]). Scores on the OSS-3 were categorized into poor (0 = scores 3–8), moderate (1 = scores 9–11), and strong (2 = scores 12–14) [50]. We also used three predictors from T2: *number of family members*, *temporarily laid off* (yes/no), and *reduced income* (yes/no)—the latter two with reference to the COVID-19 pandemic.

### Analytic strategy

We applied LPA and LTA analyses using Mplus 8.5 to address our research questions. LPA is a person-centred approach involving the formation of profiles (i.e. subgroups/classes) based on patterns of similarities among individuals on the indicator variables. For a general overview, see Lanza, Bray, and Collins [51]. Before we ran LTA, we examined potential latent QoL profiles at each time point with 500 starting values and 20 for final optimization [52]. We selected the number of LPAs in line with recommendations [53], mainly focussing on the bootstrap likelihood ratio test (BLRT) and the Bayesian Information Criterion (BIC).

Following the tradition of a mixture model, we considered to model the within-class indicator covariance (i.e. relationship between variables in a given class) and allow for different variances across profiles. Yet, due to the model not converging with the relaxed parameters, we chose a more parsimonious model with the assumption of local independence and variance homoscedasticity in the form of a classic LPA/LTA. For a similar approach, see Moore et al. [37] and Stronge et al. [54]. When model fit criteria do not provide conclusive evidence for selecting the number of classes, a more parsimonious model is usually chosen [37, 55]. To examine the associations between baseline covariates (T1) and observed LPAs (T1 and T3), we applied a multinomial logit model which uses multiple predictors to model categorical outcomes. We used the 3-step approach suggested by Asparouhov and Muthén [56] to account for the measurement error in class classification.

Based on the LPAs identified at each wave, we applied LTA. This is a longitudinal extension of LPA, estimating the probability of transitioning from one class to another over time [57]. The LTA process began with the identified profiles at each assessment. The observed LPAs at T were then regressed on the profiles from T1. First, we tested a first-order invariant LTA model specifying the transition matrices to be equal over time. This model fitted poorly in terms of AIC, BIC, and Entropy. Consequently, we did not specify a stationary transition probability across the transition period (which included the Covid outbreak). We also included a second-order effect (i.e. effects of LPAs at T1 on the LPAs T3), given the likelihood of a lasting effect of initial status. This LTA model showed better model fit. Hence, our final model was the LTA model specifying a second-order effect and freely estimated transition matrices.

We applied the 3-step approach to avoid the potential bias that the classification of LPAs at *T* is affected by an observed measurement at *T* − 1 in the conventional 1-step approach (entropy values which show the accuracy of class assignment (0 to 1) in our LPA were about 0.80 implying potential classification errors) [56]. Given the possibility of unobserved variables that are likely to confound the observed transitional patterns over time (e.g. personality, district SES), we also compared our findings with estimates from an LTA with random intercepts (RI-LTA). The RI-LTA addresses unobserved stable confounding effects using a latent variable approach [58]. We employed a maximum likelihood estimator with robust standard errors.

## Results

### Descriptives

Table 1 provides descriptive information. Overall, QoL decreased over time, particularly during the later stage (T2–T3) as indicated by the significant mean level differences of NE and LS (about 25% of a *SD*). The observed decreases in meaning levels were very small, illustrating the possibility that individuals may react differently to sub-dimensions of QoL. We also briefly explored sex differences in QoL (see Online Appendix A). Although there were some fluctuations, QoL had declined for both men and women at T3. Yet, women reported more negative experiences than men during the pandemic.

**Table 1:** Descriptive statistics of variables

Variables	Mean	%	SD	Range	*N*
QoL measures					
Negative Emotion_T1	2.8	–	2.3	0–10	8.156
Negative Emotion_T2^a^	2.7	–	2.2	0–10	8.156
Negative Emotion_T3^a^	3.3	–	2.3	0–10	8.156
Positive Emotion_T1	7.0	–	1.6	0–10	8.156
Positive Emotion_T2^a^	6.9	–	1.7	0–10	8.156
Positive Emotion_T3^a^	6.7	–	1.7	0–10	8.156
Life satisfaction_T1	7.8	–	1.9	0–10	8.156
Life satisfaction_T2	7.7	–	1.9	0–10	8.156
Life satisfaction_T3^a^	7.2	–	2.0	0–10	8.156
Meaning of life_T1	7.8	–	2.0	0–10	8.156
Meaning of life_T2^a^	7.7	–	2.0	0–10	8.156
Meaning of life_T3	7.6	–	2.9	0–10	8.156
Baseline covariates					
Male	–	46.6		0–1	8.156
Age	54.1	–	14.0	20–92	8.156
Subjective income	3.7	–	1.2	0–5	7.889
Primary school	–	10.4		0–1	8.139
High school	–	33.6		0–1	8.139
College 2–3 years	–	24.9		0–1	8.139
College over 4 years	–	31.0		0–1	8.139
Single	–	18.7		0–1	8.149
Married/cohabiting	–	75.4		0–1	8.149
Non-resident partner	–	5.9		0–1	8.149
Employed	–	67.7		0–1	8.156
Other works	–	25.1		0–1	8.156
Unemployed	–	14.1		0–1	8.156
Subjective health	2.8	–	0.8	0–4	8..149
Oslo Support Scale	1.4	–	0.7	0–2	8.116
Trust	7.5	–	2.2	0–10	8.147
Belonging	7.7	–	2.4	0–10	8.149
Laid off	–	6.2		0–10	8.156
Reduced income	–	11.9		0–10	8.156
Number of family member	2.5	–	1.2	1–10	8.067

^a^A significant mean difference between *T* and *T* − 1 measures

### Latent QoL profiles before and after the COVID-19 outbreak

Before we conducted the LTA, we explored the latent QoL profiles at each assessment. Table 2 presents the model fit indices for the identified LPA structures. At time 1 and 3, the BLRT results favoured a 5-class solution, accepting the null model of 5 classes in favour of 6 at *p* < 0.001. In terms of BIC, a 6-class specification had the smallest value at all assessments. This specification just added one similar profile with a small number of observations (*n* = 159) and had a model convergence issue, despite increased random starting values. We thus selected the 5-class solution for our preferred LPA at each wave.

**Table 2:** Model fit comparisons of LPA

	Classes	Parms	LL	Entropy	AIC	Model fit		BLRT
						BIC	ssBIC	
Time 1	1	8	− 46,291.451	N/A	92,598.901	92,654.953	92,629.531	N/A
	2	13	− 40,048.531	0.91	80,123.062	80,214.146	80,172.835	C1 vs. C2***
	3	18	− 37,691.066	0.83	75,418.133	75,544.250	75,487.050	C2 vs. C3***
	4	23	− 36,694.307	0.83	73,434.614	73,595.763	73,522.674	C3 vs. C4***
	5	28	− 36,291.668	0.81	72,639.335	72,835.517	72,746.539	C4 vs. C5***
	6	33	− 35,965.771	0.80	71,997.541	72,228.756	72,123.889	C5 vs. C6
Time 2	1	8	− 46,291.451	N/A	92,598.901	92,654.953	92,629.531	N/A
	2	13	− 40,843.951	0.88	81,713.901	81,804.986	81,763.674	C1 vs. C2***
	3	18	− 38,813.164	0.81	77,662.328	77,788.445	77,731.245	C2 vs. C3***
	4	23	− 37,913.150	0.82	75,872.300	76,033.450	75,960.360	C3 vs. C4***
	5	28	− 37,621.309	0.79	75,298.617	75,494.800	75,405.821	C4 vs. C5***
	6	33	− 37,376.632	0.80	74,819.264	75,050.478	74,945.611	C5 vs. C6***
Time 3	1	8	− 46,291.451	N/A	92,598.901	92,654.953	92,629.531	N/A
	2	13	− 40,950.774	0.87	81,927.548	82,018.633	81,977.321	C1 vs. C2***
	3	18	− 39,034.622	0.80	78,105.244	78,231.361	78,174.160	C2 vs. C3***
	4	23	− 38,313.712	0.78	76,673.425	76,673.425	76,761.485	C3 vs. C4***
	5	28	− 38,041.106	0.77	76,138.213	76,334.395	76,245.416	C4 vs. C5***
	6	33	− 37,874.541	0.78	75,815.082	76,046.297	75,941.429	C5 vs. C6

*N* = 8156

*Parms* parameters, *AIC* Akaike’s Information Criterion, *BIC* Bayes’ information Criterion, *CAIC* consistent AIC, *ssBIC* sample size adjusted BIC, *BLRT* bootstrapped likelihood ratio test

In general, the model with smaller values of ICs is preferred. A higher value of Entropy implies that there are fewer errors in classification of latent profiles

*** p < .001

Table 3 provides the estimated means (standardized) and observed LPAs before the COVID-19 outbreak. Based on the observed patterns, we labelled the five emerging classes as *Troubled* (very high NE and very low PE, LS, and meaning—pre-pandemic prevalence 4.7%), *Languishing* (high NE and low PE, LS, and meaning—14.3%), *Content-Symptomatic* (moderate levels of LS, meaning, and PE, but high NE—10.6%), *Content* (moderate levels of LS, meaning, PE, and NE—31.2%), and *Flourishing* (high LS, Meaning, and PE and low NE—39.3%).

**Table 3:** Observed characteristics of latent profiles at time 1

Latent profiles	*N*	Items	Mean (SE)	Variance (SE)
Troubled	378	Life satisfaction	− 2.70 (0.07)	0.25 (0.01)
		Meaning in life	− 2.51 (0.09)	0.31 (0.01)
		Negative emotion	1.62 (0.06)	0.33 (0.01)
		Positive emotion	− 2.16 (0.06)	0.39 (0.01)
Languishing	1159	Life satisfaction	− 1.14 (0.06)	0.25 (0.01)
		Meaning in life	− 1.13 (0.05)	0.31 (0.01)
		Negative emotion	0.95 (0.03)	0.33 (0.01)
		Positive emotion	− 1.07 (0.04)	0.39 (0.01)
Content-symptomatic	823	Life satisfaction	0.07 (0.05)	0.25 (0.01)
		Meaning in life	0.26 (0.04)	0.31 (0.01)
		Negative emotion	1.31 (0.05)	0.33 (0.01)
		Positive emotion	− .06 (0.06)	0.39 (0.01)
Content	2560	Life satisfaction	− .05 (0.02)	0.25 (0.01)
		Meaning in life	− 10 (0.03)	0.31 (0.01)
		Negative emotion	− .16 (0.04)	0.33 (0.01)
		Positive emotion	− .11 (0.03)	0.39 (0.01)
Flourishing	3236	Life satisfaction	0.76 (0.02)	0.25 (0.01)
		Meaning in life	0.72 (0.01)	0.31 (0.01)
		Negative emotion	− .76 (0.01)	0.33 (0.01)
		Positive emotion	0.75 (0.02)	0.39 (0.01)

Variables are standardized. The local independence and variance homoscedasticity are assumed in LPA

### Factors associated with the latent profiles before COVID-19

As shown in Table 4, men were more likely to belong to the *troubled* than the *flourishing* group (OR = 1.4), while women were more likely to belong to the *content-symptomatic*. Individuals in the oldest age group had systematically higher odds of belonging to the *flourishing* group, while single and those reporting low income had systematically lower odds. Educational levels were also significantly related to belonging to the *troubled* and *content* groups. College education significantly increased the odds of belonging to the *content* over the *flourishing* group. Compared to primary school, secondary and college (2–3 years) education were associated with higher odds of belonging to the *troubled* over the *flourishing* group. Lower levels of self-rated health, social support (OSS-3), interpersonal trust, and sense of belonging to the local community were systematically associated with lower odds of belonging to the flourishing group.

**Table 4:** Multinomial logistic model using the 3-step approach (ref = flourishing) at time 1

Variables	Classification											
	Troubled			Languishing			Content-symptomatic			Content		
	*b*	SE	OR	*b*	SE	OR	*b*	SE	OR	*b*	SE	OR
Male	0.34*	0.17	1.40	− 0.06	0.11	0.94	− 0.40***	0.11	0.67	0.21*	0.09	1.23
Age	− 0.05***	0.01	0.95	− 0.03***	0.01	0.97	− 0.02**	0.01	0.98	− 0.02***	0.00	0.98
Subjective income	− 0.43***	0.07	0.65	− 0.32***	0.05	0.73	− 0.23***	0.05	0.79	− 0.11**	0.04	0.89
Primary school (ref)												
High school	0.57*	0.26	1.76	0.16	0.19	1.18	− 0.18	0.18	0.84	0.32	0.17	1.38
College 2–3 years	0.82**	0.28	2.27	0.35	0.20	1.42	− 0.37	0.20	0.69	0.55**	0.18	1.73
College over 4 years	0.55	0.29	1.73	0.18	0.19	1.20	− 0.14	0.19	0.87	0.52**	0.18	1.68
Single (ref)												
Married/cohabiting	− 1.34***	0.19	0.26	− 1.02***	0.14	0.36	− 0.16	0.16	0.85	− 0.70***	0.12	0.50
Non-resident partner	− 0.82*	0.35	0.44	− 0.59*	0.25	0.55	− 0.20	0.27	0.82	− 0.58**	0.22	0.56
Employed (ref)												
Other (disabled, retired)	− 0.23	0.25	0.79	0.04	0.14	1.05	− 0.41**	0.16	0.67	0.11	0.12	1.11
Unemployed	0.53*	0.23	1.70	0.34	0.18	1.41	− 0.01	0.20	0.99	0.23	0.16	1.26
Subjective health	− 2.34***	0.13	0.10	− 1.64***	0.09	0.20	− 0.70***	0.08	0.50	− 0.87***	0.07	0.42
Social support	− 1.89***	0.15	0.15	− 1.39***	0.10	0.25	− 0.74***	0.10	0.48	− 0.80***	0.08	0.45
Trust	− 0.50***	0.04	0.61	− 0.37***	0.03	0.69	− 0.25***	0.03	0.78	− 0.21***	0.03	0.81
Belonging	− 0.63***	0.04	0.53	− 0.48***	0.03	0.62	− 0.25***	0.03	0.78	− 0.31***	0.03	0.74

Latent profiles and predictors are from the baseline assessment (T1)

*p < 0.05; **p < 0.01; ***p < 0.001. *N* = 7813

Table 5 shows the results from the multinomial logit model predicting the observed patterns of class membership at T3. In general, the observed magnitudes of the associations were reduced. At T3 (November/December 2020), women were consistently less likely to belong to the *flourishing* group than men. Notably, we still observed that baseline higher levels of subjective income, self-rated health, perceived social support, interpersonal trust, and sense of belonging were related to lower odds of belonging to all non-referenced groups (vs. flourishing group). Of the three T2 covariates (last three rows), reduced income due to the pandemic was significantly associated with higher odds of belonging to the *content-symptomatic* group over the *flourishing* group, while increased number of family members was associated with lower odds.

**Table 5:** Multinomial logistic model using the 3-step approach (ref = flourishing) at time 3

Variables	Classification											
	Troubled			Languishing			Content-symptomatic			Content		
	*b*	SE	OR	*b*	SE	OR	*b*	SE	OR	*b*	SE	OR
Male	− 0.53*	0.22	0.59	− 0.69***	0.10	0.51	− 0.85***	0.13	0.43	− 0.50***	0.09	0.61
Age	− 0.05***	0.01	0.95	− 0.01**	0.01	0.99	− 0.03***	0.01	0.97	− 0.01	0.00	0.99
Subjective income	− 0.45***	0.09	0.64	− 0.19***	0.05	0.82	− 0.28***	0.06	0.75	− 0.14**	0.05	0.87
Primary school (ref)												
High school	− 0.09	0.30	0.91	0.26	0.18	1.30	0.00	0.21	1.00	0.22	16	1.25
College 2–3 years	0.31	0.33	1.37	0.40*	0.18	1.49	0.17	0.22	1.18	0.35*	0.16	1.42
College over 4 years	− 0.60	0.41	0.55	0.47**	0.18	1.60	0.15	0.22	1.16	0.39*	0.16	1.47
Single (ref)												
Married/cohabiting	− 0.82**	0.27	0.44	− 0.56***	0.15	0.57	− 0.64**	0.20	0.53	− 0.18	0.15	0.84
Non-resident partner	− 0.17	0.37	0.84	− 0.45	0.23	0.64	− 0.50	0.29	0.60	− 0.18	0.22	0.84
Employed (ref)												
Other works	0.62*	0.29	1.86	0.31*	0.13	1.36	0.52**	0.17	1.69	− 0.01	0.12	0.99
Unemployed	0.38	0.27	1.46	0.02	0.17	1.01	0.18	0.20	1.20	− 0.20	0.17	0.83
Subjective health	− 1.45***	0.15	0.23	− 0.98***	0.07	0.38	− 1.38***	0.10	0.25	− 0.62***	0.07	0.54
Oslo support scale	− 1.07***	0.20	0.35	− 0.70***	0.09	0.50	− 0.95***	0.11	0.39	− 0.36***	0.09	0.70
Trust	− 0.40***	0.06	0.67	− 0.23***	0.03	0.80	− 0.26***	0.04	0.77	− 0.17***	0.04	0.84
Belonging	− 0.37***	0.05	0.69	− 0.27***	0.04	0.77	− 0.35***	0.04	0.71	− 0.20**	0.04	0.82
Laid off	0.45	0.42	1.56	0.33	0.23	1.39	0.29	0.29	1.33	− 0.01	0.22	0.99
Reduced income	0.50	0.32	1.64	0.17	0.17	1.19	0.54*	0.22	1.72	0.16	0.16	1.18
Number of family members	− 0.13	10	0.88	− 0.04	0.05	0.97	− 0.15*	0.07	0.86	− 0.05	0.05	0.95

*p < 0.05; **p < 0.01; ***p < 0.001. *N* = 7729

### Latent transitional patterns of QoL after the COVID-19 outbreak

Table 6 shows the class proportions for each time point from our baseline LTA model and how the observed proportions changed over time. Specifically, the pre-pandemic proportions of individuals assigned to the *content* and *flourishing* groups were about 71%. The proportion of individuals classified into the *troubled* group gradually decreased from time 1 (5%) to time 3 (2%). Decreasing proportions were also observed for the *flourishing* and *content-symptomatic* groups (16 and 2 percentage points [pp]). In contrast, the proportions of individuals in the *languishing* and *content* groups increased by 6 and 15 pp, respectively. The most dramatic (relative) change (i.e. 40% and 60%) thus occurred for the flourishing and troubled groups.

**Table 6:** Percentages of individuals in each latent profile between time 1 and time 3

Class	Time 1	Time 2	Time 3
Troubled	5	3	2
Languishing	14	17	20
Content-symptomatic	10	8	8
Content	31	42	46
Flourishing	40	31	24

Table 7 provides the transitional probabilities after the COVID-19 outbreak with the 3-step LTA, controlling for the second-order effect. As shown in Table 7, we observed strong stability in the *flourishing* (67%) and *content* (65%) groups after the COVID-19 outbreak. Individuals in the two main “at-risk” groups showed less stability with estimates of 43% (languishing) and 36% (troubled). Notably, the observed stabilities became more pronounced between T2 and 3. The transitional probabilities of the *troubled* and *languishing* groups increased to 49% and 57%, respectively. Only 1% of those classified into the *troubled* group at T2 moved to the *flourishing* group. For those in the *flourishing* group at T2, 4% moved to the *languishing* and *content-symptomatic* groups. Greatest stability was observed for the *content* group. This pattern implies that the most disadvantaged and advantaged groups at the outset were highly likely to remain in their groups also during COVID-19. However, substantial, mostly unfavourable, transitions occurred.

**Table 7:** Transitional probabilities for change in profile membership from the 3-step LTA

Time	Class	Troubled	Languishing	Content-SMC	Content	Flourishing
T1–T2	Troubled	**0.36**	0.15	0.42	0.06	0.00
	Languishing	0.01	**0.43**	0.31	0.49	0.21
	Content-SMC	0.04	0.25	**0.04**	0.19	0.03
	Content	0.01	0.25	0.04	**0.65**	0.06
	Flourishing	0.00	0.01	0.01	0.31	**0.67**
T2–T3	Troubled	**0.49**	0.15	0.36	0.00	0.01
	Languishing	0.07	**0.57**	0.15	0.12	0.01
	Content-SMC	0.01	0.36	**0.44**	0.26	0.02
	Content	0.00	0.16	0.02	**0.75**	0.07
	Flourishing	0.00	0.03	0.01	0.26	**0.71**

*T* time

Bold refers to observed stability over time for the given classes/profiles

## Discussion

In this study, we examined QoL changes, subgroup differences in QoL profiles, their predictors, and transition patterns in a large Norwegian community sample amid COVID-19. Overall, QoL levels decreased during the pandemic. We identified five unique QoL profiles at all assessments, namely *troubled, languishing, content-symptomatic, content*, and *flourishing*. The proportions belonging to these groups varied widely, from 2–5% in the *troubled* group to 24–40% in the *flourishing* group. Overall, the subgroups differed more in their QoL levels than in their configurations. However, the presence of a *content-symptomatic* class characterized by fairly high levels of both wellbeing and distress indicated that traditional “bipolar” measures of mental health may not capture the complexity of psychological reactions sufficiently. Hence, our findings underscore the value of “dual factor” [59] or “dual-continua” [60] QoL classes with different trajectories and socio-emotional outcomes over time [37].

An important contribution of our study is the assessment of change over time in terms of transitions between holistic QoL classes during COVID-19. Overall, we find that the pandemic has made a clear shift in both QoL levels and the distribution of QoL profiles in Norway. The well-known characteristics of the Norwegian society were reflected in the large pre-pandemic proportion of individuals in the *content* and *flourishing* groups (71%). From before the pandemic to 9 months into the pandemic, we observed considerable changes, particularly towards the *content* and *languishing* groups, whose proportions increased by 48% and 43%, respectively, while the *flourishing* and *troubled* groups decreased by 40% and 60%. The latter finding runs counter to our initial expectations. Thus, the COVID-19 pandemic seems associated with some shift from the more extreme towards the more moderate groups, thus “equalizing” the QoL distribution at least temporarily. This is in line with the findings from one large variable-centred Norwegian adolescent study [61]. Another study of Norwegian adolescents reported that youth from low socio-economic backgrounds showed more adverse changes in psychosocial outcomes (i.e. no equalizing effect) during the pandemic [62]. In line with our findings based on adults, this large-scale Norwegian study also underscored substantial stability, which is likely to reflect genetic influences [22, 25] shared with personality, most notably extraversion and neuroticism [41, 63] and stable environmental factors.

In terms of transition probabilities, strong stability was observed for the most advantaged (flourishing) and disadvantaged (troubled) groups. During the pandemic (T2–T3), the observed stabilities of these two groups were 71% and 49%, respectively, despite mean levels of the individual QoL measures dropping substantially during this period. Corresponding estimates for the remaining subgroups varied from 44% (content-symptomatic) to 75% (content). A similar but less stable pattern was also evident in the period comprising the outbreak (T1–T2). As a supplemental analysis, we also explored potential sex differences in these transitional patterns and observed broadly similar results. We also conducted a sensitivity test using the recently developed RI-LTA to account for unobserved heterogeneity in the transition process (see Online Appendix B). Similar patterns were observed, with stability becoming stronger over time, providing a degree of confidence in our finding of substantial stability.

Our results are thus in line with the previous findings of notable changes in QoL in response to major events, collective stressors, and macrolevel changes followed by adaptation [64, 65]. Greater stability for the *flourishing* (i.e. “multi-asset”) group suggests that accumulation of socio-economic and psychological assets provides individual or circumstantial resources needed to mitigate adverse development. Pre-pandemic poor health and income, weak social integration, younger age, and being single increased the odds of belonging to the *troubled* group and may contribute to cement an adverse situation.

The strong links between wellbeing, psychopathology, and social relationships are evidenced by numerous sources. The majority of people show prosocial tendencies from an early age and derive benefits of prosocial action [28, 66]. High-quality relations and social support promote health and wellbeing as well as resilience to stressors [67], and communities with higher social capital appear to rebound faster after natural disasters such as earthquakes and tsunamis [68, 69]. Our findings highlight the importance of both strong (social support) and weak (trust, belonging) social bonds along with the importance of economic and health-related factors to QoL, as these factors were strongly associated with the disadvantaged classes at the outset.

Our study has several strengths, including its prospective design, range of QoL items, and large sample size. Another noteworthy strongpoint is the use of robust person-centred methods addressing unobserved heterogeneity and the configuration of key QoL variables permitting us to examine mixed and holistic emotional reactions. Some caveats should also be noted. First, panel samples tend to over-represent those with higher socio-economic status, and access to a computer or internet-connected mobile phone was necessary to complete the survey. Compromised health, lower education, financial difficulties, and younger age significantly predicted drop-out from T1 in our study. Consequently, those most affected by the pandemic and associated measures (i.e. troubled) are somewhat under-represented in our sample. In ancillary analyses, we ran the analysis with data from all participants (i.e. not only those participating at all assessments). The identified latent profiles using both approaches were highly similar. We note, however, that the means estimated for our outcome variables, and the proportion belonging to the flourishing class at T1, were modestly lower in the full sample. As associations, but not means, are relatively robust against non-random missing [70, 71], attrition in our study may compromise the external validity of our means and prevalence estimates. Second, the study is limited by its reliance on self-report. Two measures consisted of single items, and the internal consistency of the PE measure was also less than optimal (0.71). Third, we selected a parsimonious model specifying local independence and variance homoscedasticity, partly due to a model convergence issue. Future studies with a larger sample size may need to consider various types of measurement structures to provide richer information on related items and latent profiles. Fourth, reactions to the pandemic will be context-dependent, and the results may thus not extrapolate to other contexts, regions, and nations. The Nordic welfare states are known for their universalistic approach to welfare and introduced significant measures to safeguard jobs after the outbreak. Trust in the government is high, and the infectious levels and mortality rate from COVID-19 have been rather modest, perhaps contributing to lower the level of concerns. However, the prevalence of subsyndromal anxiety and depression was estimated to 18% in a nationally representative Norwegian sample (*N* = 17,000) during the initial lockdown [29], and not substantially different from average international estimates ranging between 16 and 28% [72]. Fifth, social relations are likely to have changed during the pandemic. Optimally, social support should have been included at T2 and T3 and future studies need to investigate this in further detail. Lastly, our study stretches from 1 to 5 months before until 9 months into the pandemic, with the second assessment conducted in June 2020, when the infectious measures were less strict. Hence, there might be unobserved time-varying factors including seasonal effects.

The course of the pandemic is unknown, as are the long-term effects on societies and individuals. Future studies are therefore needed to investigate trajectories of QoL onwards and in different settings. Given the likely recurrence of COVID-19, future pandemics, social unrest, and natural disasters, there is a need to establish evidence-based knowledge about viable wellbeing, promotion of social integration, coping, and adaptation. Our study yields useful information with respect to theories on adaptation and factors that support or challenge QoL which are relevant also beyond the pandemic.

## Supplementary Information

Below is the link to the electronic supplementary material.

Supplementary file1 (DOCX 35 kb)
