# Supplementary material for: Flattening the quality of life curve? A prospective person-centred study from Norway amid COVID-19
Source: Qual Life Res. 2022 Mar 24;31(8):2295–305. doi: 10.1007/s11136-022-03113-2 (PMC8942803; doi:10.1007/s11136-022-03113-2)
Supplement: Supplementary file 1 — Supplementary file1 (DOCX 35 kb) [file 11136_2022_3113_MOESM1_ESM.docx]

| **Variables** | **Male**  (n=3,799) | | | **Female**  (n=4,357) | | **Mean Diff** |
| --- | --- | --- | --- | --- | --- | --- |
|  | Mean | SD | Mean | | SD |  |
| Negative Emotion_T1 | 2.6 | 2.2 | 3.1 | | 2.2 | -.52** |
| Negative Emotion_T2 | 2.4 | 2.1 | 3.0 | | 2.2 | -.54** |
| Negative Emotion_T3 | 2.9 | 2.2 | 3.7 | | 2.3 | -.73** |
| Positive Emotion_T1 | 7.0 | 1.6 | 7.0 | | 1.6 | .03 |
| Positive Emotion_T2 | 7.1 | 1.6 | 6.8 | | 1.7 | .21** |
| Positive Emotion_T3 | 6.8 | 1.7 | 6.6 | | 1.8 | .27** |
| Life satisfaction_T1 | 7.8 | 1.9 | 7.7 | | 1.9 | .05 |
| Life satisfaction_T2 | 7.8 | 1.9 | 7.7 | | 1.9 | .10* |
| Life satisfaction_T3 | 7.4 | 1.9 | 7.1 | | 1.9 | .35** |
| Meaning of life_T1 | 7.7 | 2.0 | 7.8 | | 2.0 | -.07 |
| Meaning of life_T2 | 6.9 | 1.8 | 6.9 | | 1.8 | .05 |
| Meaning of life_T3 | 7.6 | 1.9 | 7.6 | | 2.0 | .04 |

**Appendix A**. Sex differences in QoL measures

*Note.* * *p* < .01, * *p* < .001. Independent t-test result is reported.

**Appendix B.** Transitional probabilities for change in profile membership from the RI-LTA

| Time | Class | Troubled | Languishing | Content-SMC | Content | Flourishing |
| --- | --- | --- | --- | --- | --- | --- |
| T1-T2 | Troubled | **.66** | .00 | .04 | .01 | .29 |
|  | Languishing | .05 | **.47** | .12 | .11 | .35 |
|  | Content-SMC | .01 | .02 | **.47** | .35 | .04 |
|  | Content | .03 | .08 | .38 | **.40** | .12 |
|  | Flourishing | .11 | .00 | .22 | .04 | **.64** |
| T2-T3 | Troubled | **.84** | .00 | .03 | .01 | .12 |
|  | Languishing | .01 | **.51** | .10 | .14 | .21 |
|  | Content-SMC | .00 | .01 | **.62** | .36 | .03 |
|  | Content | .02 | .07 | .34 | **.51** | .07 |
|  | Flourishing | .07 | .00 | .14 | .02 | **.78** |

*Note.* T denotes Time.
